# Fungal-mediated synthesis of silver nanoparticles: a novel strategy for plant disease management

**DOI:** 10.3389/fmicb.2024.1399331

**Published:** 2024-06-28

**Authors:** Mansoor Ahmad Malik, Abdul Hamid Wani, Mohd Yaqub Bhat, Sazada Siddiqui, Saad A. M. Alamri, Sulaiman A. Alrumman

**Affiliations:** ^1^Section of Plant Pathology and Mycology Laboratory, Department of Botany, University of Kashmir, Srinagar, India; ^2^Department of Biology, College of Science, King Khalid University, Abha, Saudi Arabia

**Keywords:** antimycotic, antifungal, *Cladosporium cladosporioides*, *Rhizoctonia solani*, silver nanoparticles

## Abstract

Various traditional management techniques are employed to control plant diseases caused by bacteria and fungi. However, due to their drawbacks and adverse environmental effects, there is a shift toward employing more eco-friendly methods that are less harmful to the environment and human health. The main aim of the study was to biosynthesize silver Nanoparticles (AgNPs) from *Rhizoctonia solani* and *Cladosporium cladosporioides* using a green approach and to test the antimycotic activity of these biosynthesized AgNPs against a variety of pathogenic fungi. The characterization of samples was done by using UV–visible spectroscopy, SEM (scanning electron microscopy), FTIR (fourier transmission infrared spectroscopy), and XRD (X-ray diffractometry). During the study, the presence of strong plasmon absorbance bands at 420 and 450 nm confirmed the AgNPs biosynthesis by the fungi *Rhizoctonia solani* and *Cladosporium cladosporioides*. The biosynthesized AgNPs were 80–100 nm in size, asymmetrical in shape and became spherical to sub-spherical when aggregated. Assessment of the antifungal activity of the silver nanoparticles against various plant pathogenic fungi was carried out by agar well diffusion assay. Different concentration of AgNPs, 5 mg/mL 10 mg/mL and 15 mg/mL were tested to know the inhibitory effect of fungal plant pathogens *viz*. *Aspergillus flavus, Penicillium citrinum, Fusarium oxysporum, Fusarium metavorans,* and *Aspergillus aflatoxiformans*. However, 15 mg/mL concentration of the AgNPs showed excellent inhibitory activity against all tested fungal pathogens. Thus, the obtained results clearly suggest that silver nanoparticles may have important applications in controlling various plant diseases caused by fungi.

## Introduction

Nanotechnology exploration has recently sparked significant interest within the domain of material sciences. It stands out as one of the latest breakthroughs imbued with novelty and quintessentially addresses the emerging challenges our world faces. In the pharmaceutical and biomedical industries, nanoparticles exhibit a diverse range of applications, including gene and drug delivery systems, water disinfection, electronics, biosensors, as well as serving as agents for anticancer, antibacterial, antifungal, and antiprotozoal purposes (Lee and Jun, 2019; Rai et al., 2021; War et al., 2022). In recent years, there has been significant research interest in the biosynthesis and characterization of nanoparticles. This interest is primarily driven by their large surface area, which imparts unique properties and potential applications distinct from their bulk counterparts (Shnoudeh et al., 2019; Sajid and Płotka-Wasylka, 2020). Up to now, nanoparticles have been synthesized using a variety of chemical and physical techniques. However, biosynthesis approach utilizing biological systems such as plants, fungi, yeast, and bacteria has been explored and adopted globally for nanoparticle biosynthesis due to its environmentally friendly, reproducible, non-toxic, and cost-effective nature (Gudikandula et al., 2017; Hano and Abbasi, 2021).The biosynthesis approach offers many advantages over chemical synthesis (Duan et al., 2015; Roy et al., 2019; Murali et al., 2023). Since biological systems operate as natural reducing, stabilizing, and capping agents, thereby avoiding many processes for the synthesis of nanoparticles, which not only decreases the cost and consumption of chemicals, but also eliminates agglomeration and oxidation of synthesized nanoparticles (Sidhu et al., 2022). Metallic nanoparticles, especially silver nanoparticles (AgNPs) produced through biological sources, have undergone intensive investigation as a potential alternative therapy for a wide range of infections and illnesses (Bhuyan et al., 2017; Al-Ansari et al., 2020; Murali et al., 2021; Nandi et al., 2023).

Silver is widely known to combat certain microorganisms by modifying their cell membrane structure and function (Dakal et al., 2016; Mikhailova, 2020; Das and Dutta, 2021). Silver is employed as a disinfectant in water purification systems due to its ability to destroy bacteria at low concentrations (less than 1–10 m) (Liu et al., 1994; Deshmukh et al., 2019; Bhardwaj et al., 2021; Das and Dutta, 2022). However, silver can be hazardous to animals, freshwater, and marine organisms at greater quantities (Tortella et al., 2020). Interestingly, micromolar quantities of silver are not toxic to humans (Vrček et al., 2016). Therefore, silver has been widely used in the production of various biological and medicinal products. Recent studies revealed that the toxicity of Ag-NPs measured in freshwater depends on the test species (Blinova et al., 2013). For example, Ag-NPs are reported to be toxic for crustaceans at very low concentration (EC_50_ < 0.1 mg L^−1^), followed by algae (EC_50_ = 0.23 mg L^−1^), but the toxicity to fish is relatively low (EC_50_ = 7.1 mg L^−1^, Kahru and Dubourguier, 2010; Asghari et al., 2012). It has been observed that effects of AgNP on the T84 epithelial cells were size- and dose-dependent, with the 10 nm AgNP causing the most significant changes. Changes in permeability of the epithelial cell monolayer, as measured by transepithelial electrical resistance, after exposure to 10 nm AgNP were most dramatic at the highest dose (100 μg/mL), but also observed at the lower dose (20 μg/mL) (Williams et al., 2016).

Presently, AgNPs are widely utilized across a broad spectrum of applications, ranging from electronic devices to biological tools (Garg et al., 2020; Tufail and Liaqat, 2021; Naganthran et al., 2022). This is likely due to the stability of the particles, which holds significant importance for various applications, particularly in medicine. Additionally, it is crucial that nanoparticles do not agglomerate during their formation, to achieve enhanced stability, maximal yield, and controlled size aggregation of particles, optimization of the various parameters employed in plant-mediated nanoparticle biosynthesis is essential (Ebrahiminezhad et al., 2018; Ahmad et al., 2024). Recent reports have demonstrated the broad-spectrum antibacterial activity of AgNPs against both Gram-positive and Gram-negative bacteria, including multidrug-resistant strains (Liao et al., 2019; Ansari et al., 2021; Yassin et al., 2022). It is significant that AgNPs exhibit multiple modes of inhibitory action against microorganisms, as opposed to the single specific action of antibiotics (Vazquez-Muñoz et al., 2019). Interestingly, AgNPs demonstrate efficacy against various fungi, including *Candida* spp., dermatophytes, and certain phytopathogenic fungi such as *Bipolaris sorokiniana* and *Magnaporthe grisea* (Rajeshkumar, 2019; Mansoor et al., 2021). Conversely, various phytopathogenic fungi remain unexplored, despite their role in causing severe diseases in crucial crop plants, consequently diminishing agricultural yield. Therefore, the present study aimed to address the following questions: (a) to study the preparation and characterization of silver nanoparticles. (b) To study the efficacy of various concentrations of biosynthesized silver nanoparticles against fungal pathogens.

## Materials and methods

### Fungal culture

In this study, *Rhizoctonia solani* and *Cladosporium cladosporioides* were isolated from soil samples. Pure cultures of these fungi were cultivated on Potato Dextrose Agar and identified based on cultural, morphological, and microscopic characteristics as described by Trappe (1982), Quimio (2001), Boerema et al. (2004).

### Preparation of silver nanoparticles

**Figure 1:**
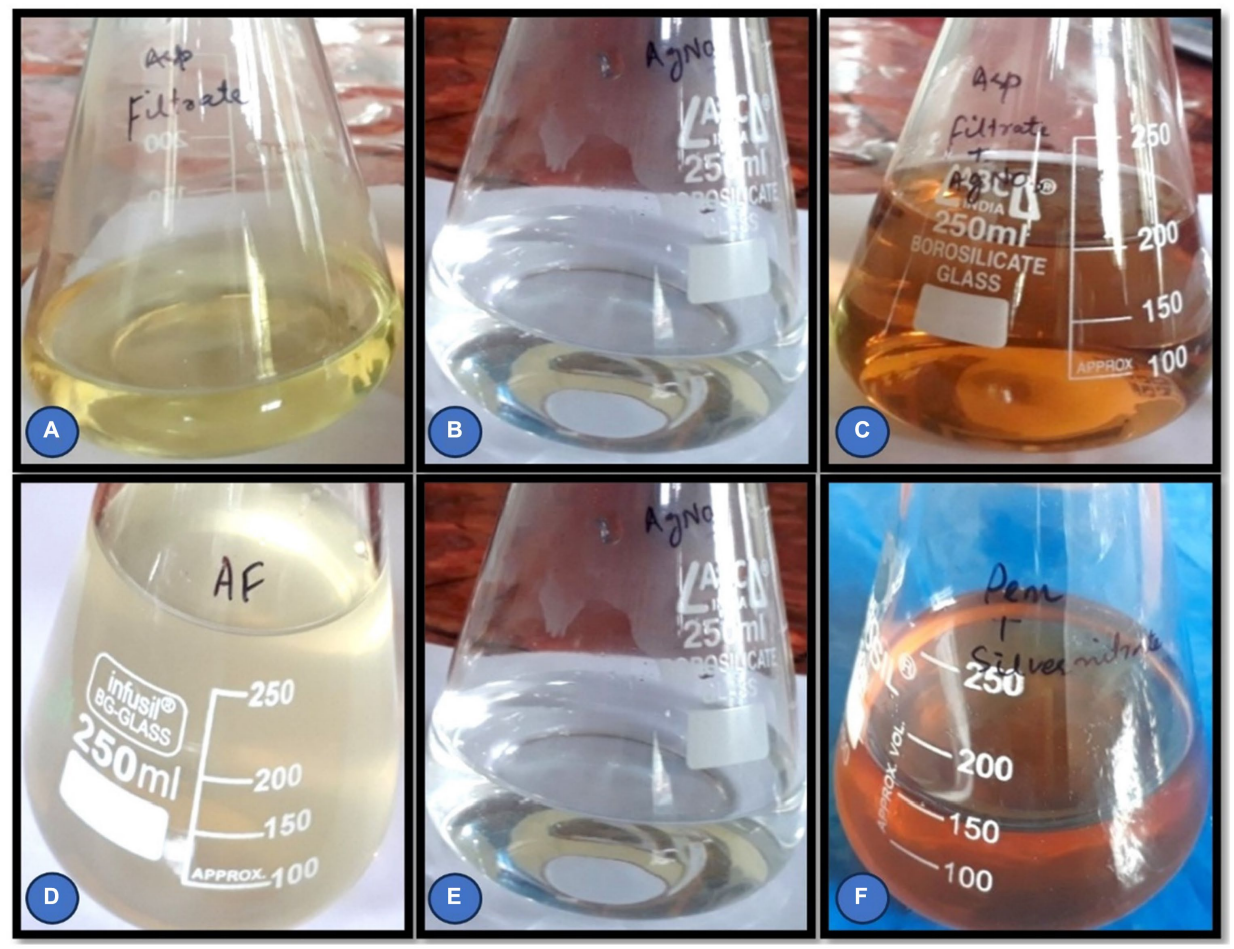
**(A–F)** Cell filtrate of *Rhizoctonia solani* and *Cladosporium cladosporioides* before and after the addition of AgNO_3_ solution.

To biosynthesize AgNPs the fungi *Rhizoctonia solani* and *Cladosporium cladosporioides* were cultured separately in 250 mL conical flasks, each containing 100 mL of potato dextrose broth. The flasks were then incubated at 26 ± 2°C for 72-96 h. Then mycelial mat separation was performed using Whatman filter paper. The medium components were eliminated from the biomass by washing with double-distilled water 3–4 times. Approximately 25 g of fresh biomass was placed in 250 mL conical flasks containing 200 mL of double-distilled water and left for 24–72 h at 25°C. Subsequently, 50 mL of cell filtrate was combined with 50 mL of AgNO_3_ silver nitrate solution. Solution and the reaction mixture was kept in orbital shaker at 37^0^c and 200 rpm for 24 h. The conversion of Ag ions to (AgNPs) was confirmed by color change from yellow to brown. A reaction mixture without AgNO_3_ served as a control and was kept alongside the experimental flasks (Sagar and Ashok, 2012; Talie et al., 2020) (Figures 1A–F).

### Characterization of silver nanoparticles

Different methods were employed to characterize the biosynthesized AgNPs.

#### Color change

The reduction of silver ions was routinely monitored visually over a period 24 h. The key presence of a brown color in the reaction mixture is an indicator of the formation of silver nanoparticles. The color change is caused by the activation of surface plasmon vibrations.

#### UV–visible spectroscopy analysis

Ultraviolet (UV) spectroscopy confirms the formation of silver nanoparticles by reducing silver nitrate. The UV–Vis spectra of the cell filtrate was recorded after 24 h on a UV–Visible absorption spectrophotometer (UV–visible Spectrophotometer 119, SYSTRONICS) with a resolution of 2.0 nm between wavelengths of 350–700 nm possessing a scanning speed of 300 nm/min.

#### Scanning electron microscopy (SEM)

Scanning electron microscopic analysis was used to measure the size and shape of silver nanoparticles. For SEM, (AgNPs) were biosynthesized using cell filtrate, normally required to be completely dry and the specimen was dried and grounded to a powder.

#### Fourier transmission infrared spectroscopy (FTIR)

The suspension of AgNPs biosynthesized using *Rhizoctonia solani* and *Cladosporium cladosporioides* was centrifuged at 10,000 rpm for 20 min at room temperature. The resulting residue was washed several times with sterile distilled water, dried in 40°C and finally the AgNPs were stored in vials. The collected powdered AgNPs were then taken for FTIR analysis in the range of 450–4,500 cm^-1^.

#### X-ray diffractometry (XRD)

The AgNPs solution obtained after bio-reduction was purified by centrifugation at 10,000 rpm for 20 min, followed by redispersion of the AgNPs pellet into 1 mL of sterile de-ionized water. X-ray diffraction (XRD) was used to examine the structure and content of the purified AgNPs after they were freeze dried. The dried mixture of AgNPs was collected for X-ray diffractometer analysis of AgNPs production. The colloidal suspensions of AgNPs were analyzed by XRD to validate their crystalline nature in order to verify the results of the UV spectral analyses.

#### Antifungal efficacy of biosynthesized silver nanoparticles against some selected fungal pathogens

Effect of biosynthesized AgNPs on the mycelial growth of some selected soil pathogenic fungi was analyzed. The agar well diffusion experiment was performed to investigate the antifungal activity of biosynthesized AgNPs against fungal pathogens such as *Aspergillus flavus*, *Penicillium citrinum*, *Fusarium oxysporum*, *Fusarium metavorans,* and *Aspergillus aflatoxiformans*. An aliquot of 0.02 mL of inoculum of each test fungal pathogen was injected into culture tubes containing 20 mL of molten Sabouraud dextrose agar medium. The culture tubes were homogenized and then emptied into petri plates and allowed to harden under laminar airflow chamber (aseptic conditions). A 5 mm conventional cork borer was used to make wells on the agar plate. Three different concentrations *viz*. 05, 10, and 15 mg/mL of AgNPs were prepared and 50 μL from each concentration was added to respective wells. Nystatin 50 μL/disc was utilized (positive control) as a control. The effect of AgNPs on the test fungal infections was analyzed and compared to the reference standard. The antifungal activity was determined using the standard scale of Norrel and Messley (1997).

## Results

Plant pathogens such as bacteria and fungi are controlled by different traditional management strategies. However, some of these management measures, such as the use of pesticides, have negative environmental consequences due to their limits. As a result, alternative ways are used that are more environmentally friendly and have fewer negative consequences on human health and the environment. As a result, the antimycotic activity of various AgNPs was tested against fungal pathogens in this work. These AgNPs were prepared by simple technique and used at different concentrations against pathogenic fungi. AgNPs were biosynthesized using *Rhizoctonia solani* and *Cladosporium cladosporioides*. After preparation of these AgNPs they were processed to prepare different concentrations. These different concentrations of AgNPs were screened for their antifungal activity against fungal pathogens.

### Characterization of biosynthesized silver nanoparticles using fungi

Different techniques employed for the characterization of biosynthesized AgNPs are given below:

#### Color change

The Initial indication of AgNPs biosynthesis was confirmed by the color change. The color of *Rhizoctonia solani* and *Cladosporium cladosporioides* fungal filtrates changes from colorless to brown, as seen in the results (Figures 1A–F). Change in color of the cell free filtrate incubated with silver nitrate solution was visually exhibited after 24 h of incubation which clearly indicates the AgNPs formation.

#### UV–visible spectroscopy analysis

**Figure 2:**
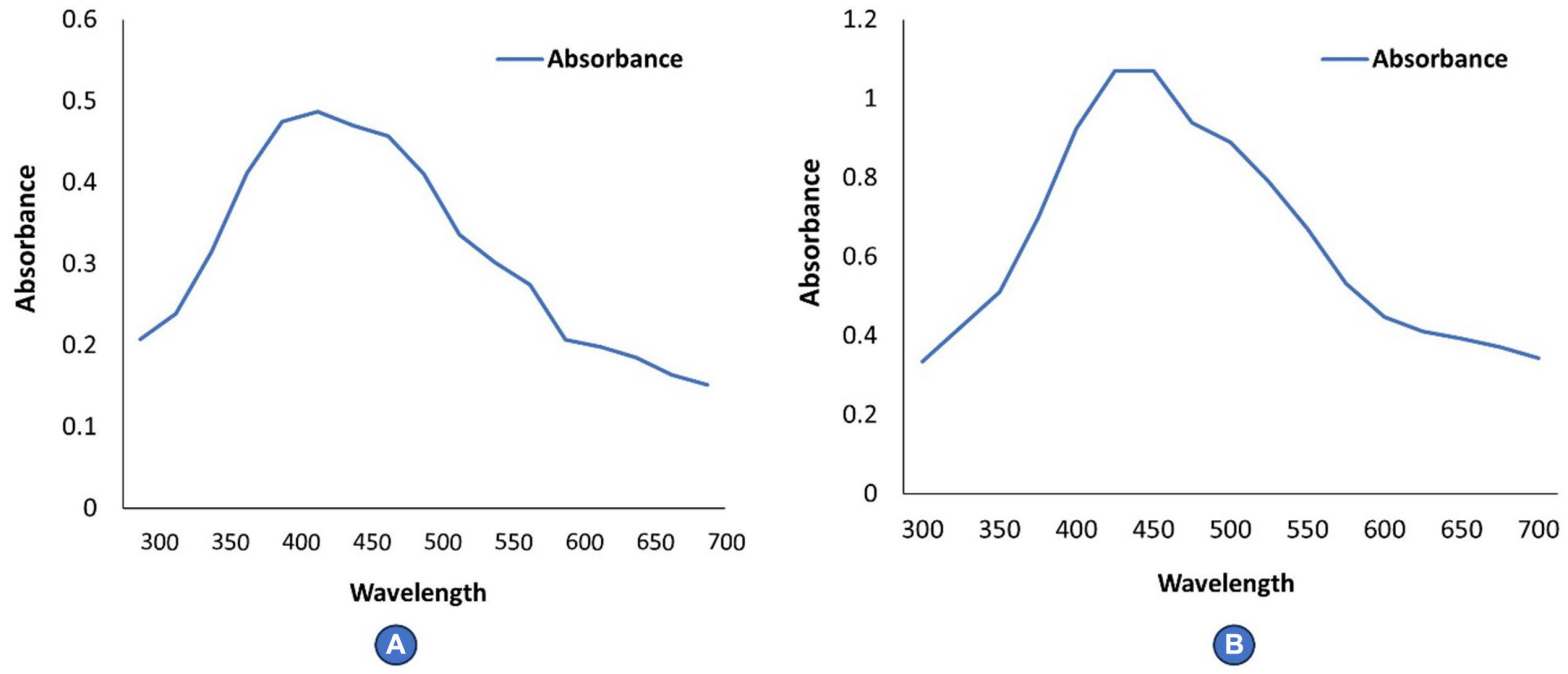
**(A,B)** Depicts UV–Vis spectra of AgNPs.

The reduction of silver nitrate to AgNPs was confirmed by ultraviolet (UV) Spectroscopy. The UV–Visible spectra of *Rhizoctonia solani* and *Cladosporium cladosporioides* cell filtrates showed strong plasmon absorption bands at 425 and 450 nm (Figures 2A,B).

#### Scanning electron microscopy (SEM)

**Figure 3:**
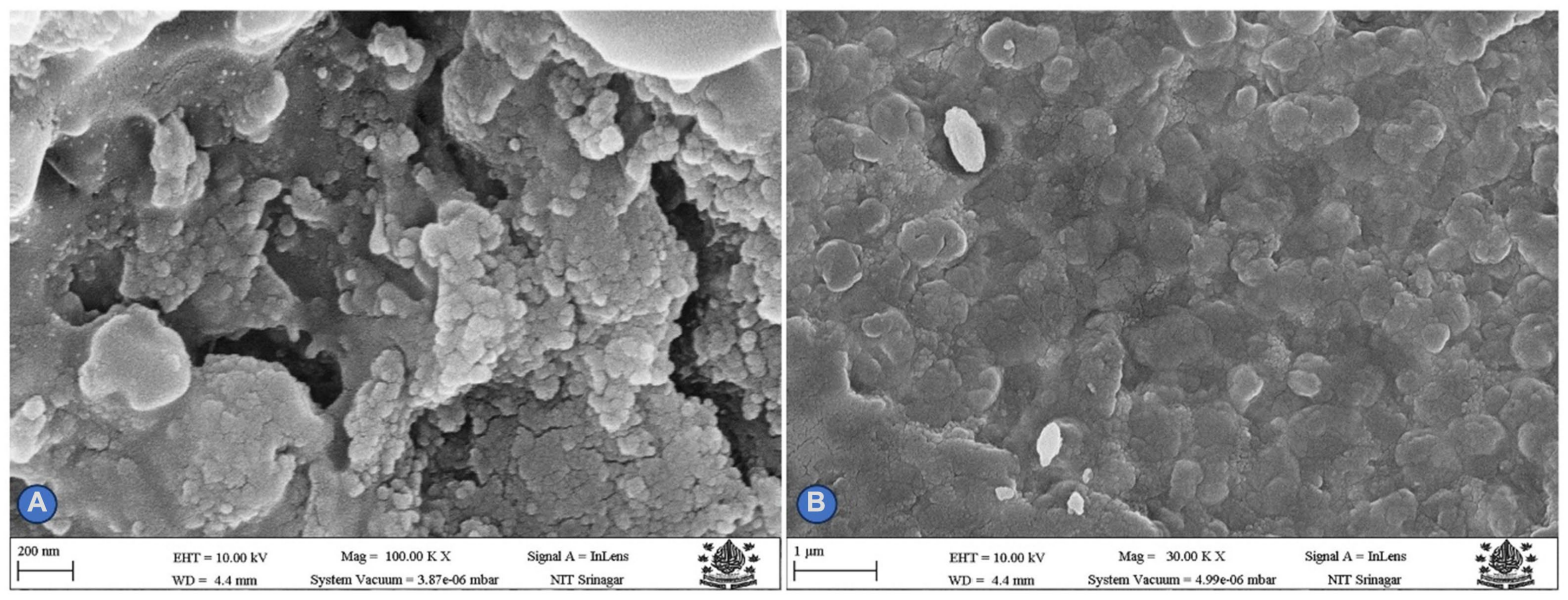
**(A,B)** Depicts SEM micrograph of AgNPs.

The mean particle size and shape of biosynthesized AgNPs were studied using scanning electron microscopy. SEM pictures of silver nanoparticles synthesized from *Rhizoctonia solani* and *Cladosporium cladosporioides* are shown in Figures 3A,B. The aggregated shape of biosynthesized AgNPs was found to be irregular and spherical. AgNPs range in size from 80 to 100 nanometers.

#### Fourier transmission infrared spectroscopy (FTIR)

**Figure 4:**
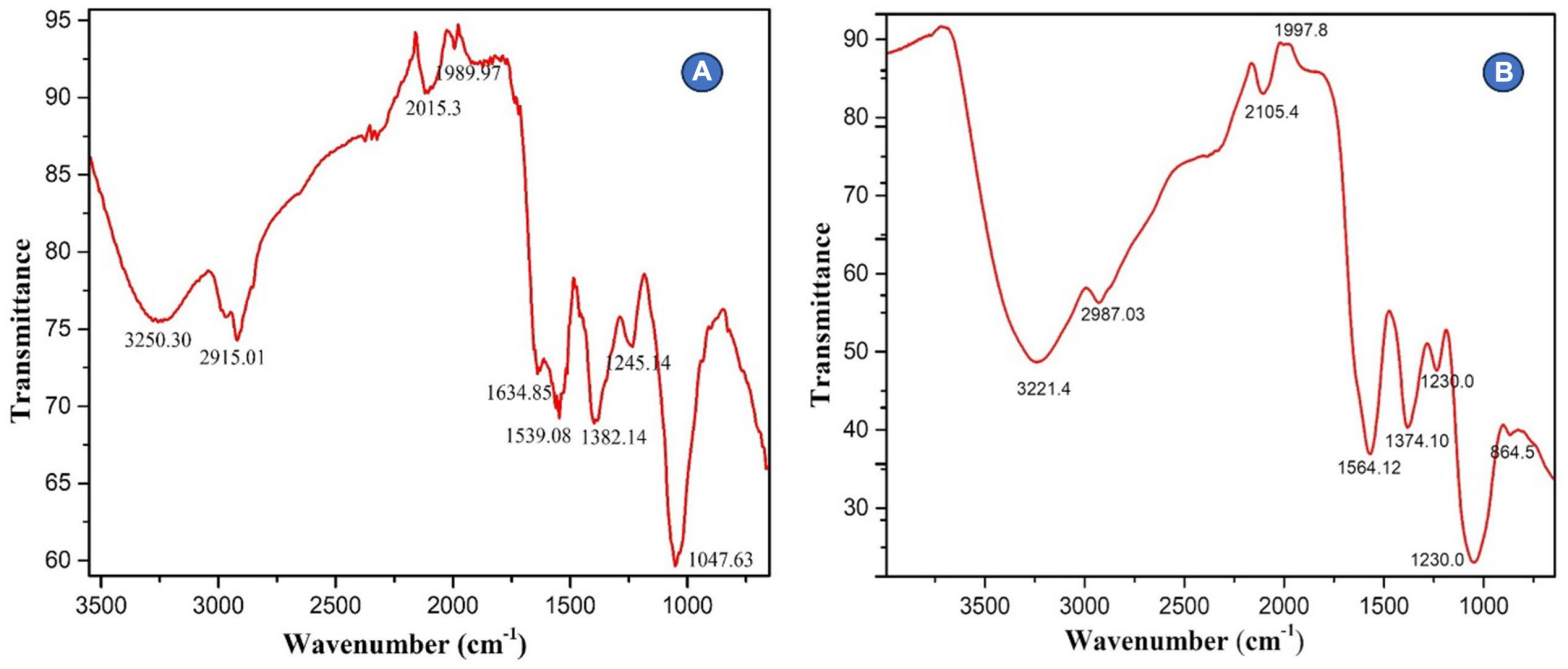
**(A,B)** Depicts FTIR spectroscopy of silver nanoparticles.

The FTIR spectroscopy was used to examine biosynthesized AgNPs in the range 450–4,500 cm^−1^. AgNPs made from *Rhizoctonia solani* were found to absorb substantially at various wavelengths (3250.30, 2915.01, 2015.3, 1989.97, 1634.85, 1539.08, 1382.14, 1245.14, and 1047.63 cm^−1^) respectively. While the absorption bands 2915.01 are related to the -OH of carboxylic acid, the absorption band 3250.30 cm^−1^ is related to the N-H amine stretch. Similar to this, the absorption bands at 1382.14 and 1634.85 cm^−1^ are related to the C-N stretching vibrations of aromatic amines and the unsaturated nitrogen molecules O-NO2 and nitrate, respectively. Furthermore, the infrared (IR) spectra show bands that reveal the existence of O-H carboxylic acid, N-H amine linkages, C-N aromatic amine linkages, and O-NO2 unsaturated nitrogen compounds, which may be present in AgNPs as stabilizing caps alongside proteins and amino acid residues. Likewise, the results revealed that biosynthesized AgNPs from fungus *Cladosporium cladosporioides* absorb strongly 3221.4, 2987.03, 2105.4, 1997.8, 1564.12, 1374.10, 1230.0, and 864.5 cm^−1^, respectively, (Figures 4A,B).

#### XRD analysis

**Figure 5:**
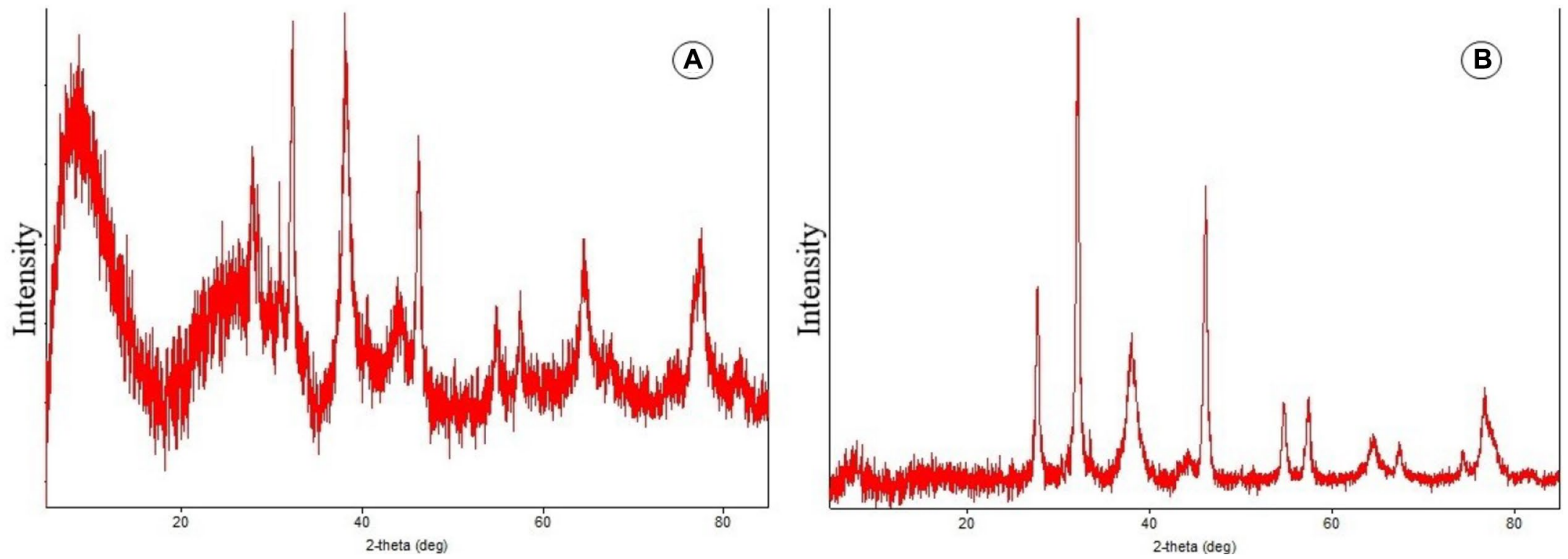
**(A,B)** Depicts X-ray diffraction pattern of AgNPs.

During the present study, the crystal structure and particle size of the biosynthesized AgNPs were determined using X-ray diffraction. Figures 5A,B represents the XRD pattern of AgNPs and biosynthesized AgNPs showed excellent crystal quality. Reference data from ICSD (inorganic crystal structure database) and ICDD (international center for diffraction data) were used and matched with data obtained during the present study using PDXL-2 software. Peaks were seen over the whole spectrum, which ranged from 20 to90 nm demonstrating the achievement of great purity. The XRD (X-ray diffraction) spectrum demonstrated that the biosynthesized AgNPs were in the form of nano crystals when compared to the reference data.

#### Antifungal activity of biosynthesized silver nanoparticles on the mycelial growth of some pathogenic fungi employing agar well diffusion method

The findings revealed that AgNPs derived from *Rhizoctonia solani* and *Cladosporium cladosporioides* at various concentrations (05, 10, and 15 mg/mL) inhibited all of the fungal pathogens such as *Aspergillus flavus, Penicillium citrinum, Fusarium oxysporum, Fusarium metavorans,* and *Aspergillus aflatoxiformans*. However, zone of inhibition increased with the increase in concentrations of AgNPs. Furthermore, the lowest concentrations of biosynthesized AgNPs reduce the zone of inhibition against all of the tested fungal strains significantly (Table 1).

**Table 1:** Geographical location of the sampling sites.

Location	Altitude (asl)	Latitude	Longitude
Gulmarg	2,650 m	34°03′14′′ N	74°23′88′′ E
Doodhpathri	2,850 m	33°50′67′′ N	74°35′15′′ E
Drang	2,300 m	34°03′32”N	74°25′57″E
Kashmir University Botanical Garden (KUBG)	1,591 m	34°09′66′′ N	74°50′77′′E

#### Antifungal efficacy of various concentrations of biosynthesized silver nanoparticles using fungus, *Rhizoctonia solani* on the zone of mycelial growth inhibition of some pathogenic fungi

**Figure 6:**
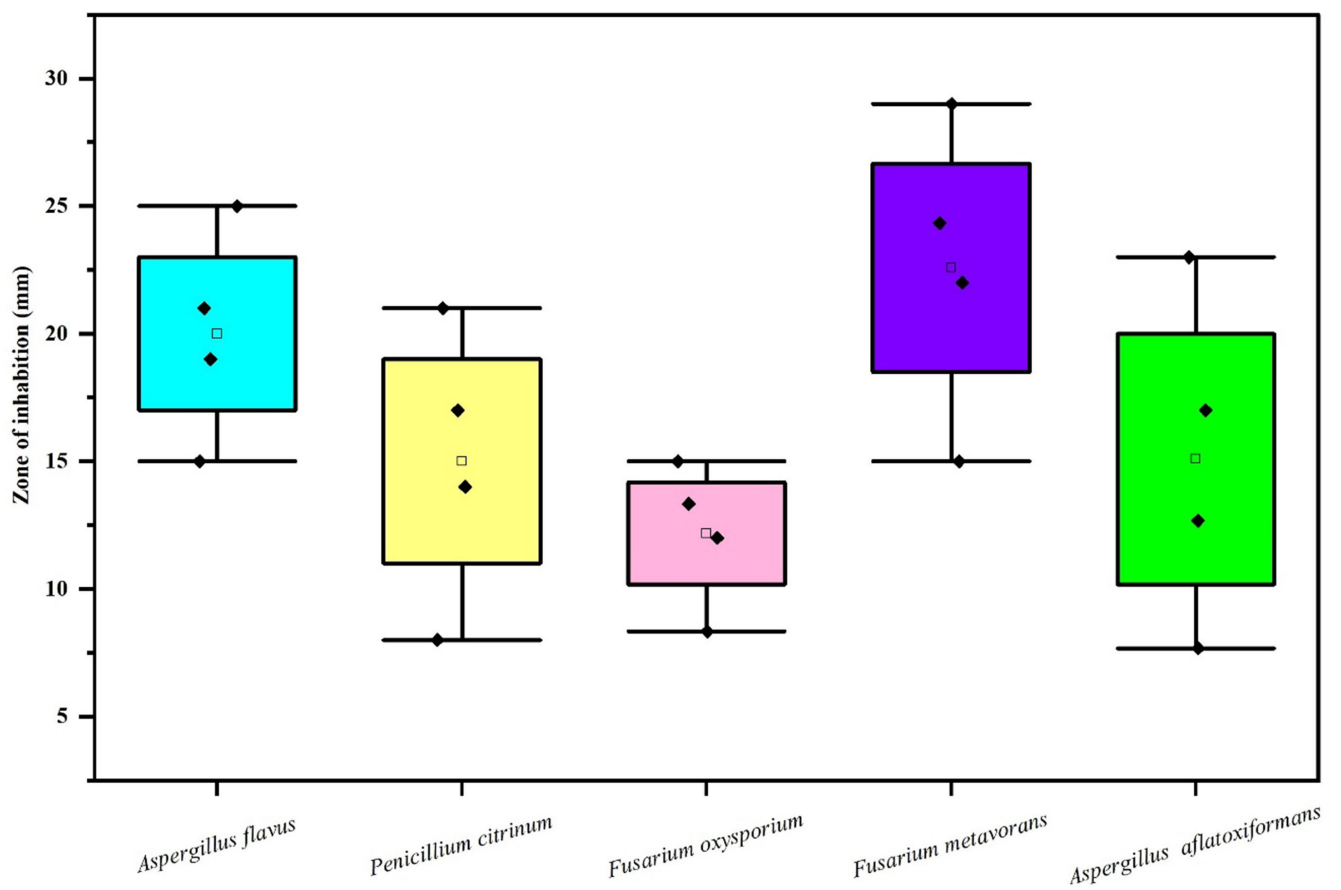
Box and whisker plot depicting the efficacy of AgNPs on zone of mycelial growth inhibition of some pathogenic fungi.

**Figure 7:**
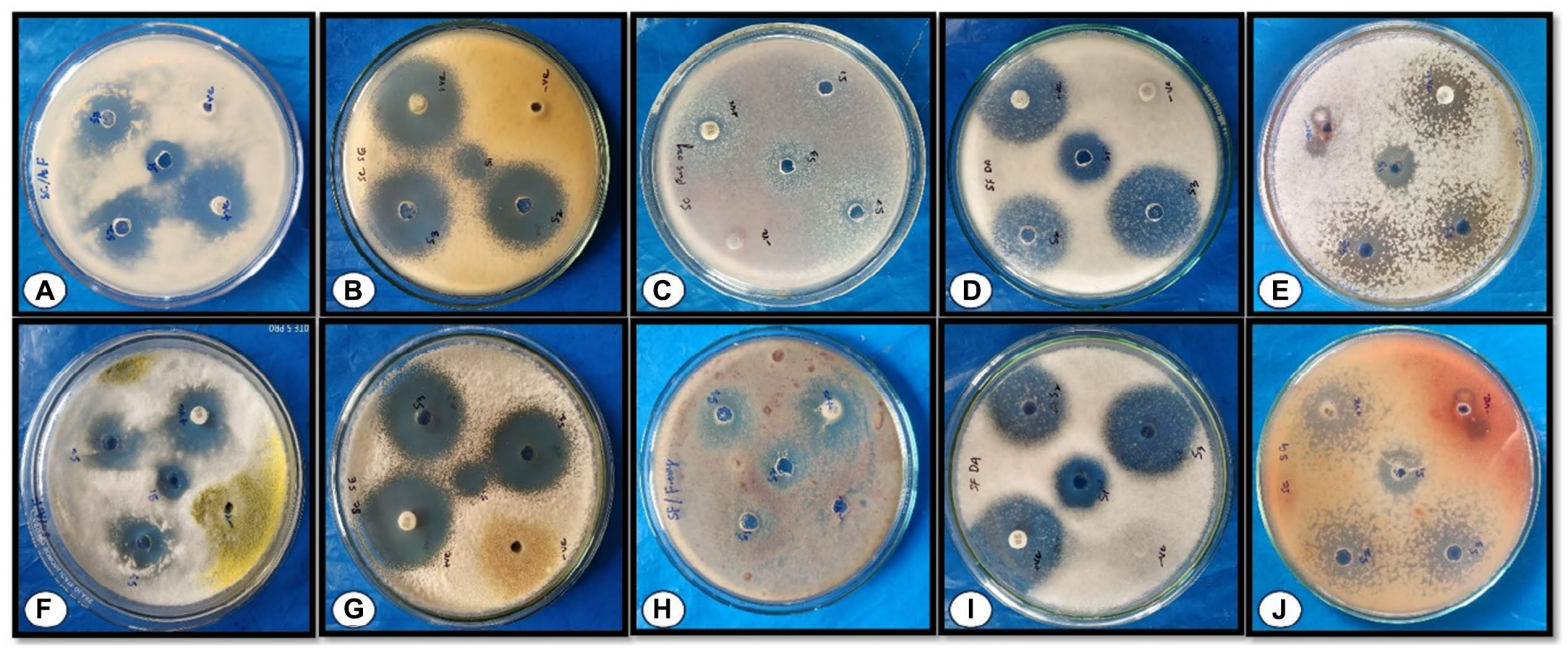
Effect of different concentrations of AgNPs on the zone of mycelial growth inhibition of some pathogens. **(A–E)** depicts the effects of AgNPs biosynthesized from *Rhizoctonia solani* **(A)** *Aspergillus flavus*, **(B)** *Pencillium citrinum*, **(C)** *Fusarium oxysporum*, **(D)** *Fusarium metavorans*, and **(E)** *Aspergillus aflatoxiformans*; **(F–J)** depicts the effect of AgNPs biosynthesized from *Cladosporium cladosporioides* **(F)** *Aspergillus flavus*, **(G)** *Pencillium citrinum*, **(H)** *Fusarium oxysporum*, **(I)** *Fusarium metavorans*, and **(J)** *Aspergillus aflatoxiformans*.

The results (Table 2 and Figures 6, 7A–E) revealed that there was found inhibition in all the tested pathogenic fungi at all the concentrations of AgNPs biosynthesized by *Rhizoctonia solani*. However, the maximum zone of inhibition against *Fusarium metavorans* (24.33 ± 0.57) was found at highest concentrations of biosynthesized AgNPs. It was followed by inhibition in mycelial growth of *Aspergillus flavus* (21.00 ± 1.00), *Penicillium citrinum* (17.00 ± 1.00), *Aspergillus aflatoxiformans* (17.00 ± 1.00), and *Fusarium oxysporum* (13.33 ± 0.57) at the same concentrations, respectively. The zone of inhibition in mycelial growth against *Aspergillus flavus* varied from 15.00 to 21.00 mm, and in case of *Penicillium citrinum* varied from 8.00 to 17.00 mm, respectively at different concentrations of AgNPs. Similarly, in case of *Fusarium oxysporum*, the zone of inhibition in mycelial growth ranges from 8.33 to 13.33 mm, for *Fusarium metavorans*, from 15.00 to 24.33 mm, and for *Aspergillus aflatoxiformans*, from 7.66 to 17.00 mm, respectively. The zone of inhibition against all the other tested fungi decreased considerably at the lowest concentrations of biosynthesized AgNPs but to lower extent.

**Table 2:** Efficacy of various concentrations of biosynthesized silver nanoparticles on the zone of mycelial growth inhibition of some pathogenic fungi.

Concentration	Zone of inhibition (mm)			
Fungal pathogens	5 mg/mL	10 mg/mL	15 mg/mL	Standard
*Aspergillus flavus*	15.00 ± 1.00^a^	19.00 ± 1.00^b^	21.00 ± 1.00^c^	25.00 ± 1.00^d^
*Penicillium citrinum*	8.00 ± 1.00^a^	14.00 ± 1.00^b^	17.00 ± 1.00^c^	21.00 ± 1.00^d^
*Fusarium oxysporum*	8.33 ± 1.52^a^	12.00 ± 1.00^b^	13.33 ± 0.57^bc^	15.00 ± 1.00^c^
*Fusarium metavorans*	15.00 ± 1.00^a^	22.00 ± 1.00^b^	24.33 ± 0.57^c^	29.00 ± 1.00^d^
*Aspergillus aflatoxiformans*	7.66 ± 0.57^a^	12.66 ± 0.57^b^	17.00 ± 1.00^c^	23.00 ± 1.00^d^

Values are represented as the mean ± SD of three replicates. Duncan’s multiple comparison test was used to compare mean values. The numbers that are followed by the similar alphabets do not differ statistically (*p* ≤ 0.05).

#### Antifungal efficacy of various concentrations of silver nanoparticles biosynthesized using fungus, *Cladosporium cladosporioides* on the zone of mycelial growth inhibition of some pathogenic fungi

**Figure 8:**
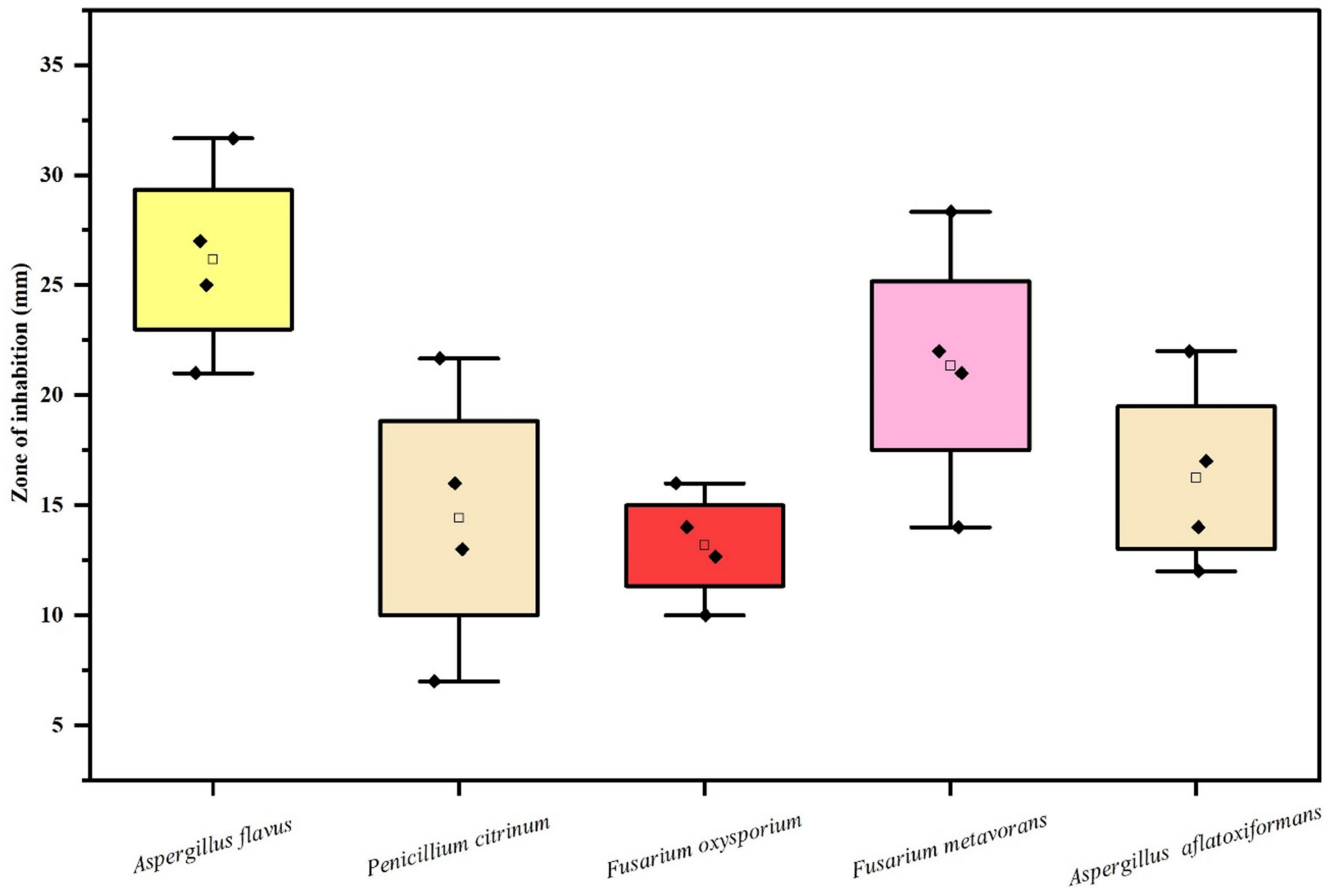
Box and whisker plot depicting the Efficacy of AgNPs on zone of mycelial growth inhibition of some pathogenic fungi.

The results (Table 3 and Figures 7F–J, 8) revealed that the AgNPs biosynthesized from *Cladosporium cladosporioides* significantly inhibited development mycelia tested fungi. However, the maximum zone of inhibition against *Aspergillus flavus* (27.00 ± 1.00) was found at the highest concentrations of AgNPs. The highest concentration of AgNPs also caused maximum reduction in the mycelial growth in case of *Fusarium metavorans* (22.00 ± 1.00), *Aspergillus aflatoxiformans* (17.00 ± 1.00), *Penicillium citrinum* (16.00 ± 1.00), and *Fusarium oxysporum* (14.00 ± 1.00) respectively. The zone of inhibition in mycelial growth against *Aspergillus flavus* varied from 21.00 to 27.00 mm, and for *Penicillium citrinum* varied from 7.00 to 16.00 mm, respectively, at different concentrations of AgNPs. Similarly, in case of *Fusarium oxysporum*, the zone of inhibition in mycelial growth ranges from 10.00 to 14.00 mm, in case of *Fusarium metavorans* it varies from 14.00 to 22.00 mm, and for *Aspergillus aflatoxiformans* varied12.00 to 17.00 mm, respectively. The zone of inhibition against all the other tested fungi also decreases considerably at the lowest concentrations of produced AgNPs.

**Table 3:** Efficacy of various concentrations of biosynthesized silver nanoparticles on the zone of mycelial growth inhibition of some pathogenic fungi.

Concentration	Zone of inhibition (mm)			
Fungal pathogens	5 mg/mL	10 mg/mL	15 mg/mL	Standard
*Aspergillus flavus*	21.00 ± 1.00^a^	25.00 ± 1.00^b^	27.00 ± 1.00^b^	31.66 ± 1.52^c^
*Penicillium citrinum*	7.00 ± 1.00^a^	13.00 ± 1.00^b^	16.00 ± 1.00^c^	21.00 ± 1.52^d^
*Fusarium oxysporum*	10.00 ± 1.00^a^	12.66 ± 0.57^b^	14.00 ± 1.00^b^	16.00 ± 1.00^c^
*Fusarium metavorans*	14.00 ± 1.00^a^	21.00 ± 1.00^b^	22.00 ± 1.00^b^	28.33 ± 1.57^c^
*Aspergillus aflatoxiformans*	12.00 ± 1.00^a^	14.00 ± 1.00^b^	17.00 ± 1.00^c^	22.00 ± 1.00^d^

*Values are represented as the mean ± SD of three replicates. Duncan’s multiple comparison test was used to compare mean values. The numbers that are followed by the similar alphabets do not differ statistically (*P* ≤ 0.05).

## Discussion

Nano-biotechnology is quickly developing as an important field of modern research, generating the most promising applications in medicine and agriculture in the present climate change scenario (Mariyam et al., 2023). The application of nano-biotechnology in agriculture will help in addressing and solving inherent imperfections and other complex problems in farm production with low input but with an efficient role due to their unique size (Dimkpa and Bindraban, 2017; Usman et al., 2020). Biosynthesis of AgNPs using green approach provide ecofriendly, clean and effective way out for the biosynthesis of nanoparticles. These nanoparticles differ in shape, size, chemical composition and other properties (Raj et al., 2021).

During the present study, *Rhizoctonia solani* and *Cladosporium cladosporioides* were used to biosynthesize AgNPs, which was proved by the appearance of brown color due to reduction of silver salt into AgNPs by fungal culture filtrates along with the appearance of strong plasmon absorbance bands at 420–450 nm and strong resonance peaks at 440 nm, thus confirming the biosynthesis of AgNPs as has been reported by Khan et al. (2018) and Paul et al. (2023). Many researchers have reported that microorganisms, plant extracts, and fungi can be used to biosynthesize nanoparticles through biological pathways (Koul et al., 2021). Many fungi like *Fusarium oxysporum*, *Aspergillus fumigatus, Aspergillus niger, Fusarium semitectum, Penicillium brevicompactum,* and *Cladosporium cladosporioides* have been reported to be competent enough to extracellularly biosynthesized AgNPs (Rajeshkumar and Sivapriya, 2020; Adebayo et al., 2021) respectively. Our results are in conformity with Verma et al. (2010) and Parveen et al. (2018), who also reported similar results for biosynthesis AgNPs and iron oxide nanoparticles from fungi, respectively. Fungi have many advantages for the production and biosynthesis of nanoparticles in comparison to other types of microorganisms and phyto extracts. This is because the mycelial mesh of fungi is easy to handle and withstands high flow pressure, agitation and many other conditions in bioreactors and other chambers. Scanning electron microscopy revealed that biosynthesized AgNPs were irregular and spherical in aggregate form, with a size ranging from 80 to 100 nm. Similar patterns of biosynthesized AgNPs were observed by Kathiresan et al. (2009) and Jain et al. (2011). Our findings are in accordance with the work of Al-Zubaidi et al. (2019) and Talie et al. (2020) who also used different microfungi and macrofungi for biosynthesis of AgNPs. It seems that the pattern aggregation and formation of mycosynthesized silver nanoparticles take place due to the enzymatic reduction of silver metal ions (Rajput et al., 2016). The results from the present study with regard to the bioactivity of biosynthesized AgNPs against phytopathogenic fungi revealed that at different concentrations, biosynthesized AgNPs caused a significant reduction in the fungal mycelial growth in terms of zone of inhibition against all the test fungal pathogens such as *Rhizoctonia solani* and *Cladosporium cladosporioide* indicating their strong antimycotic activity. Similar work was carried out by Abd-Elsalam et al. (2019), Ingle et al. (2020), Padhi and Behera (2021). Sulaiman et al. (2015) and reported antimycotic activity of bi-synthesized AgNPs against fungi, namely *Aspergillus niger, Penicillium chrysogenum, Fusarium culmorum,* and *Alternaria alternata*. Talie et al. (2020) also reported the potent antifungal activity of biosynthesized AgNPs using *Helvella leucopus* against *Aspergillus niger, Penicillium chrysogenum, Alternaria alternata* which is in conformity with our work. The results emphasize that these biosynthesized AgNPs will work best as nano-biopesticides, as has been reported by Paramo et al. (2020) and can be incorporated into the integrated disease management module. Antimycotic activity of AgNPs against different species of phytopathogenic fungi of some cereals was reported by Al-Askar et al. (2013) and *Candida* species by Ishida et al. (2013). Since phytopathogenic fungi are toxic due to the production of mycotoxins which can be easily adsorbed by nanoparticles and impart protection against disease as has been reported by Horky et al. (2018).

## Conclusion

This study focused on the biosynthesis of AgNPs from aqueous extract of the fungi, *Rhizoctonia solani* and *Cladosporium cladosporioides*. The biogenic method provides natural agents for reduction, capping and stabilization of AgNPs, which makes the synthesis approach much more cost-effective, non-toxic, reproducible and environmentally friendly, implying that fungi could be a good source of silver nanoparticles. Biosynthesized silver nanoparticles from *Rhizoctonia solani* and *Cladosporium cladosporioides* were crystallite in nature, with an average particle size of 10.100 nm. The study revealed strong antifungal properties of these synthesized AgNPs. A significant zone of mycelial growth inhibition by AgNPs was observed against all the test microorganisms. These silver nanoparticles could be of tremendous use in pharmaceutical industries for various biomedical purposes, as well as in food processing industries for food packaging to reduce contamination and enhance long-term storage and preservation of foods. Metal based nanoparticles such as silver nanoparticles may prove to be very beneficial in the agricultural sector, such as their use as nanopesticides. However, proper investigation into their mechanism of action and evaluating the impact on human health and the environment is required.

## Data availability statement

The original contributions presented in the study are included in the article/supplementary material, further inquiries can be directed to the corresponding author.

## Author contributions

MM: Conceptualization, Data curation, Formal analysis, Investigation, Methodology, Software, Visualization, Writing – original draft, Writing – review & editing. AW: Data curation, Project administration, Resources, Validation, Writing – review & editing. MB: Data curation, Investigation, Project administration, Writing – review & editing. SS: Funding acquisition, Writing – review & editing. SAMA: Writing – review & editing. SAA: Writing – review & editing.
